# First Study of Bacteremia Caused by *Herbaspirillum huttiense* in China: A Brief Research Report and Literature Review

**DOI:** 10.3389/fcimb.2022.882827

**Published:** 2022-06-17

**Authors:** Xiangyun Li, Xundi Bao, Guanhua Qiao, Lianzi Wang, Cuixiao Shi, Shuyi Chen, Yuanhong Xu, Meijuan Zheng, Zhongxin Wang

**Affiliations:** ^1^ Department of Laboratory Medicine, The First Affiliated Hospital of Anhui Medical University, Hefei, China; ^2^ Department of Laboratory Medicine, Anhui Chest Hospital, Hefei, China; ^3^ Departmentof Laboratory Medicine, Anhui Medical University, Hefei, China

**Keywords:** first study, *Herbaspirillum huttiense (H. huttiense)*, bacteremia, prompt identification, antibiotic sensitivity

## Abstract

Bacteremia caused by *Herbaspirillum huttiense (H. huttiense)* is relatively rare in positive blood cultures. *H. huttiense* is an opportunistic bacterium in patients with cancer and cirrhosis and has also been described in immunocompromised hosts. In this study, *H. huttiense* was isolated from a patient with repeated chest tightness and chest pain. Smears were prepared, stained, and examined by microscopy. Single colonies were analyzed by Gram staining, matrix-assisted laser desorption ionization-time-of-flight mass spectrometry (MALDI-TOF MS), 16S rRNA sequencing and Next-Generation Sequencing (NGS). Antibiotic sensitivity was assessed by agar dilution. Almost all publications on *H. huttiense* infections in the PubMed/ScienceDirect/EBSCO databases were reviewed and summarized. Blood sample culturing yielded white, gelatinous, and slightly raised colonies without hemolytic rings. The bacilli were found to be Gram-negative, and MS results showed 99.2% homology with *H. huttiense*. This was confirmed by 16S rRNA gene sequencing, phylogenetic tree analysis and NGS all of which were homologous with *H. huttiense* in GenBank. Antibiotic susceptibility tests were performed to determine the minimum inhibitory concentrations (MICs) of imipenem, meropenem, piperacillin-tazobactam, and levofloxacin. A comprehensive literature review revealed that *H. huttiense* was an emergent pathogen. After medical treatment, the patient’s body temperature returned to normal. This is the first report of bacteremia caused by *H. huttiense* in China. The findings could improve the awareness and attention of the rare pathogenic microorganisms in China.

## Introduction

*Herbaspirillum* species are non-fermenting, strictly aerobic, Gram-negative curved or helical bacilli that do not have hemolytic rings. They are motile with polar flagella, and oxidase-, urease-, and catalase-positive. *Herbaspirillum* species were first reported by Baldani in 1996 (Baldani et al., 1996; Obradovic et al., 2007; Dobritsa et al., 2010) and widely distributed in the environment. As nitrogen-fixing bacteria, *Herbaspirillum* species inhabit the roots of plants in the rhizosphere and have been found in wells and other ground water (Tayeb et al., 2008;. Gulati et al., 2011; Souza et al., 2013). *Herbaspirillum huttiense (H. huttiense)* is a member of *Herbaspirillum* species and has the same properties. Pathologically, although a number of *Herbaspirillum* species have been identified and studied, only a few *H. huttiense* infections have been reported as human pathogens. For example, Regunath et al. (2015) reported the first case of severe community-acquired pneumonia and bacteremia caused by *H. huttiense* in an immunocompetent adult in the USA and Liu et al. (2019) reported the first case of septicemia caused by *H. huttiense* in Korea (Regunath et al., 2015; Liu et al., 2019). In the present study, we provided the first report of a bacteremia case caused by *H. huttiense* in China. We analyzed the *H. huttiense* clinical isolate by MALDI-TOF MS and 16S rRNA gene sequencing and summarized the prompt identification and results of antibiotic sensitivity.

## Materials and Methods

### Isolation and Characterization of *H. huttiense*

Only one strain of *H. huttiense* was successfully isolated from positive aerobic blood cultures between January 2018 and January 2022 in the Department of Laboratory Medicine in the First Affiliated Hospital of Anhui Medical University. One drop of blood from positive blood cultures was inoculated onto Columbia blood plate medium and the bacteria were cultured aerobically at 37°C and 5% CO_2_, followed by Gram staining and identification under microscopy.

### MALDI-TOF MS Identification

MALDI-TOF MS identification was performed on a Vitek MS platform by the direct smear method in accordance with the instructions of the manufacturer. After acquiring the spectra, data were transferred to the analysis server which used software algorithms to compare the generated spectrum with the typical spectra in the scientific databases.

### 16S rRNA Sequencing and Phylogenetic Analysis

The original bacteria were purified and the genomic DNA was extracted. The forward and reverse primers used for PCR amplification of 16S rRNA gene were 27F(5’-AGAGTTTGATCATGGCTCAG-3’) and 1492R(5’-TACGGCTACCTTGTACGACTT-3’). The reaction procedure was 96°C for 3 min, 96°C for 30 s, 58°C for 30 s, 72°C for 1 min, 35 cycles, and 72°C for 10 min. The sequencing was compared with the 16S rRNA gene sequencing of known bacteria in the Genbank database. The phylogenetic tree was established using the MEGA7.0 software.

### Genome Sequencing and Data Assembly

The draft genome sequence of *H. huttiense* was analyzed by NGS. Genome sequencing was performed using the Illumina NovaSeq platform by generating paired-end libraries. Genomic DNA libraries for each isolate were prepared using the TruSeq DNA Sample Preparation Kit (Illumina). Adapter contamination was removed by AdapterRemoval v2 and the reads were filtered by SOAPec v2. The filtered reads were assembled into contigs and scaffolds using A5-miseq v20160825.

### *In Vitro* Antibiotic Sensitivity Test

All antibiotic sensitivity was tested using the agar dilution method.

### Literature Review

An electronic search was conducted in the PubMed/ScienceDirect/EBSCO databases using the key words “*Herbaspirillum huttiense*” to systematically search for almost all published literatures.

### Case Description

A 72-year-old man was admitted to our hospital and was diagnosed with coronary atherosclerotic cardiopathy, mitral and tricuspid insufficiency and lacunar infarction. The patient was hospitalized for a total of 58 days, from June 15th to August 12th, 2020. During hospitalization, the patient developed a high fever with an axillary temperature reaching 39.2°C. On laboratory investigations, the patient had a procalcitonin (PCT) level of 86.73 ng/mL, a CRP level of 88.17mg/L, a WBC count of 8.89×10^9^/L, and a neutrophil percentage of 84.50%. The blood was cultured using a BacT/Alert three dimensional automated blood culture system. Each blood culture consisted of a set of two (aerobic and anaerobic) bottles. Two sets of blood samples were collected from the patient. On July 3rd, two aerobic blood cultures were found positive after 20.8 h. The anaerobic blood cultures remained negative. Subsequent blood cultures were redone, and positive aerobic bacteria were still confirmed as *H. huttiense*. The patient was treated with meropenram and tigecycline for anti-infection. Moxifloxacin and piperacillin-tazobactam were changed when the condition of the patient improved. Finally the patient’s body temperature returned to normal and discharged from hospital when cured.

## Results

**Figure 1:**
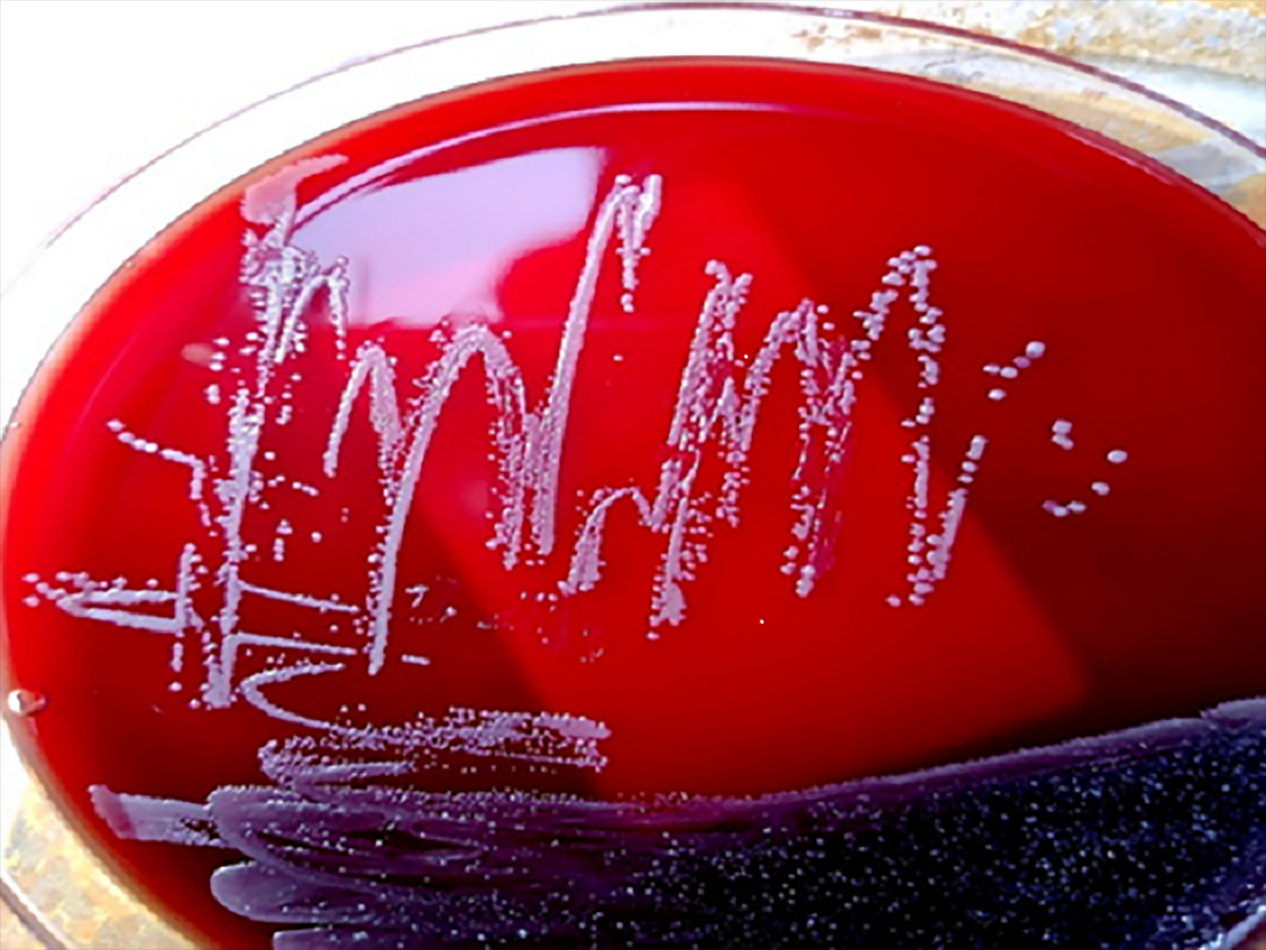
The phenotype of *H. huttiense* after 24 h aerobic culture.

**Figure 2:**
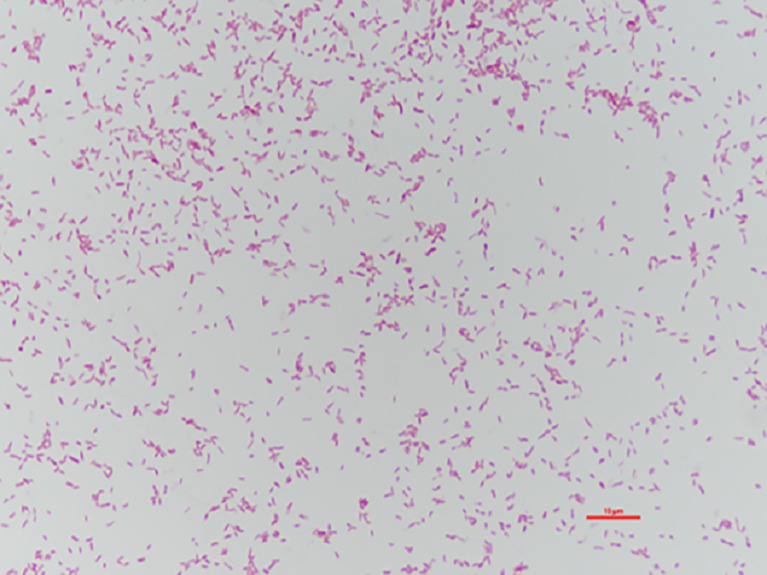
Gram-staining of *H. huttiense* showed Gram-negative bacilli.

**Figure 3:**
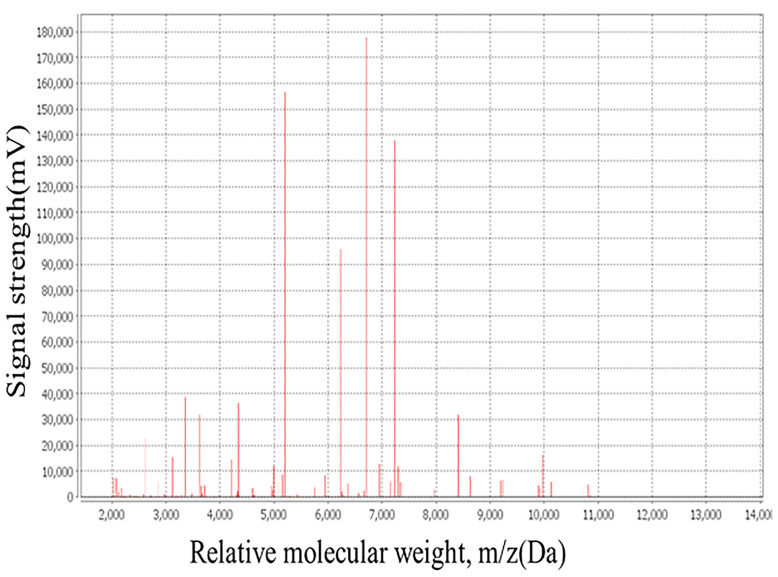
The mass spectra of *H. huttiense* revealed high accuracy.

**Figure 4:**
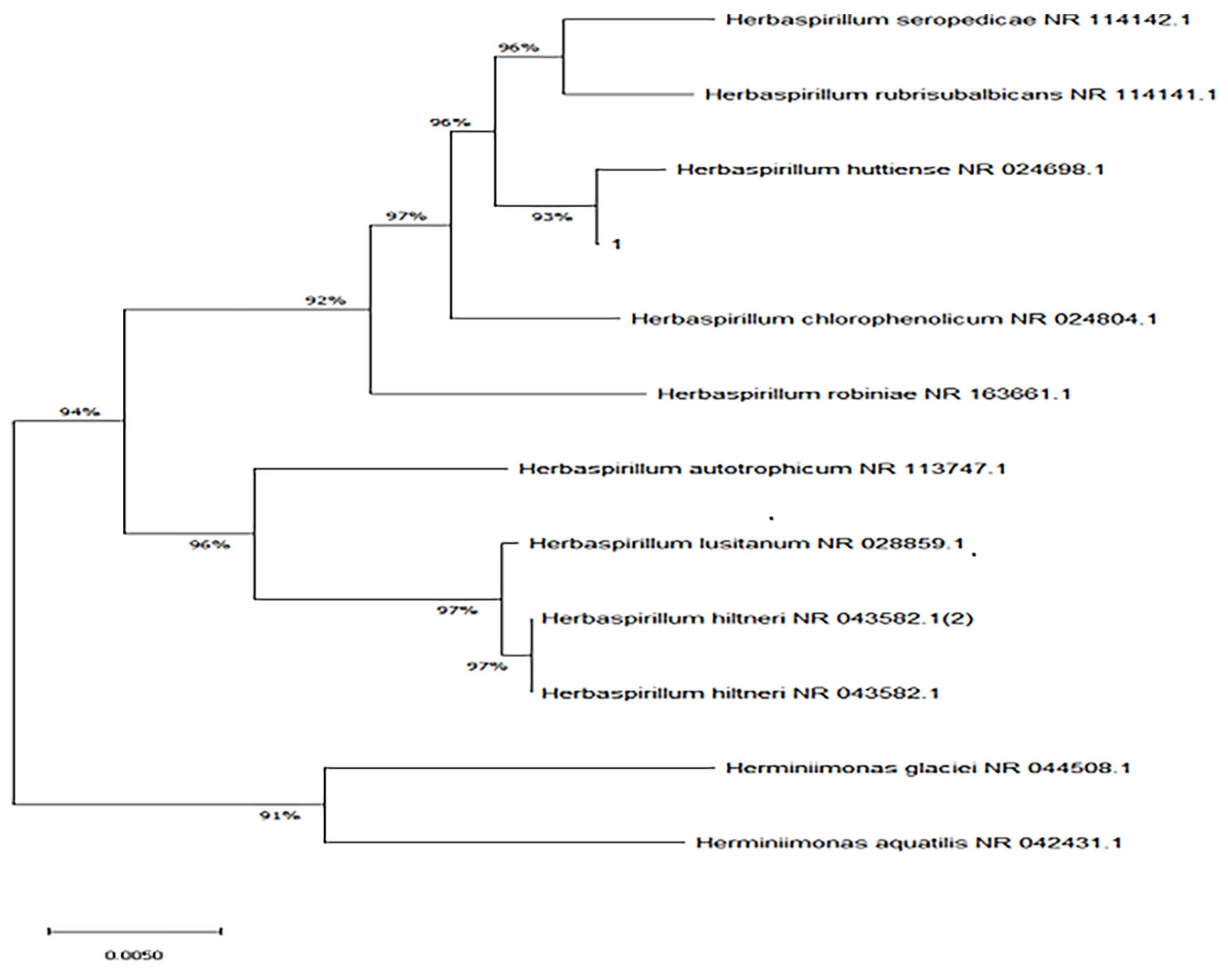
Neighbor-joining phylogenetic tree of *H. huttiense* indicated high homology (1:isolated strain).

The bacterial colonies appeared white, gelatinous, and slightly raised after 24 h in culture and had diameters between 1 and 1.5 mm without apparent hemolytic rings (**Figure 1**). Stainings showed the bacterial colonies were Gram-negative bacilli (**Figure 2**). The MALDI-TOF MS results indicated 99.2% homology with *H. huttiense* (**Figure 3**). The 16S rRNA gene sequence was consistent with that of *H. huttiense*. Phylogenetic tree analysis showed that the isolate was present on the same branch as *H. huttiense* (**Figure 4**). The isolates were thus identified as *H. huttiense* for the 16S gene. Imipenem, meropenem, piperacillin-tazobactam and levofloxacin had good antibiotic activities and the MICs results were summarized in **Table 1**. The MIC was defined as the drug concentration that completely inhibited bacterial growth or caused a marked reduction (≥90%) compared with the drug-free control. The draft genome sequence of *H. huttiense* revealed chromosome size was 5.5Mb with a 62.74% G + C content. Automatic annotation revealed 5029 open reading frames (ORFs) covering 50 virulence associated genes and 69 antibiotic resistance associated genes. Virulence associated genes including flagellum-specific ATP synthase protein, flagellar biosynthesis protein, chemotaxis regulatory protein, and purine-binding chemotaxis protein were identified in the VFDB database. Antibiotic resistance associated genes including multidrug efflux system protein, multidrug ABC transporter protein, β-lactamase protein, and DNA topoisomerase protein were detected in the CARD database. The detailed genomic features are listed in **Table 2** (Gan et al., 2020; Yang et al., 2021).

**Table 1:** MIC results of *H. huttiense*.

Antibiotics	MIC (mg/L)
Piperacillin-tazobactam	0.05
Imipenem	0.25
Meropenem	0.25
Levofloxacin	0.5

**Table 2:** Genome characteristics of *H. huttiense*.

H. huttiense				
**Genome Length**	5.5Mb			
**G+C content**	62.74%			
**Num of ORF**	5029			
**Virulence Factors of Pathogenic Bacteria (Num:50)**	Flagellum-specific ATP Synthase Protein	Flagellar Biosynthesis Protein	Chemotaxis Regulatory Protein	Purine-binding Chemotaxis Protein
**Antibiotics Resistance Genes** **(Num:69)**	Multidrug Efflux System Protein	Multidrug ABC Transporter Protein	Beta-lactamase Protein	DNA Topoisomerase Protein

After a comprehensive literature search, it was evident that *H. huttiense* was an obviously rare cause of human infections and only 8 cases of *H. huttiense* detected in human samples had been reported. These cases were mainly observed in monomicrobial infections, such as infections of pneumonia (Regunath et al., 2015; Liu et al., 2019), acute myelocytic leukemia (Nurullah et al., 2021), breast cancer (Anonymity, 2021), thrombocytopenia (Berardino et al., 2019; Anonymity, 2020), intraventricular hemorrhage (Hernández et al., 2019), and infective endocarditis (Güngör et al., 2020). Another 7 reports described *H. huttiense* in its natural habitat. As *H. huttiense* was a nitrogen-fixing bacterium and was widely distributed in the environment, it had been investigated in the roots of rice and tea plants (Gulati et al., 2011; Andreozzi et al., 2019), greenhouse tomato seedlings, pineapple and banana crops (Obradovic et al., 2007; González et al., 2019), well water and shallow ice cores (Ding and Akira, 2004; Souza et al., 2013; Chen et al., 2016). The essential data from several of the *H. huttiense* studies were summarized in **Tables 3**, **4**.

**Table 3:** Cases of *H. huttiense* infections in humans reported in the literatures.

Report year	Age/gender	Country	Diagnosis	Identicification by MALDI-TOF	Molecular investigation	AntibioticTreatment	Type of Infection
2018	93y/M	Korea	Hypotension and Pneumonia.	Bruker Biotyper(Score:2.30)	16S rRNA gene sequence	Meropenem, Colistin, Ceftazidime, Minocycline, and Trimethoprim/sulfamethoxazole	Polymicrobial
2015	46y/M	USA	Pneumonia	Bruker Biotyper	16S rRNA gene sequence	Ceftriaxone, Azithromycin, Doxycycline, Piperacillin-tazobactam	Monomicrobial
2021	54y/M	Turkey	Acute Myelocytic Leukemia	Bruker Biotyper	No	Meropenem	Monomicrobial
2018	59y/F	Spain	Thrombocythaemia and Pneumonia	MALDI-TOF MS (Brand Not Stated)	16S rRNA gene sequence	Piperacillin-tazobactam	Monomicrobial
2021	64y/F	Spain	Breast Cancer	No	No	Piperacillin/tazobactam, Ceftriaxone	Monomicrobial
2019	Two- month Old /M	Spain	Respiratory Distress and Intraventricular haemorrhage	Bruker Biotyper (more than 2.00)	No	Ceftriaxone, Cefotaxime, Meropenem	Monomicrobial
2020	Neonate [sex and exact age not stated]	USA	Thrombocytopenia	MALDI-TOF MS (Brand Not Stated)	No	Piperacillin/tazobactam	Monomicrobial
2020	11y/F	Turkey	Infective Endocarditis	Vitek MS	No	Teicoplanine, Piperacillin/tazobactom and Meropenem	Monomicrobial

**Table 4:** Cases of *H. huttiense* infections in natural habitat reported in the literatures.

Report year	Country	Natural habitat
2007	USA	greenhouse tomato seedlings
2019	France	pineapple and banana crops
2019	Italy	rice root
2011	India	tea root
2004	Japan	well water
2013	Brazil	well water
2016	China	shallow ice cores

## Discussion

A review of the literatures of *H. huttiense* infections in human showed that the ages of the patients ranged two months to 93 years old with a male-to-female ratio was 4:3. The gender ratio was balanced. The first case was described in 2015, and another 7 sporadic cases were reported since 2015. Of the eight documented cases, three (3/8) were from Spain, and two each (2/8 and 2/8) respectively came from Turkey and the United States of America. Only one (1/8) originated from Korea. Based on the world prevalence of *H. huttiense*, majority of the reported cases came from Europe, followed by America and Asia. Nonetheless, no cases were reported from Africa, and it was probable that regional differences or underdiagnosis due to lack of technical resources led to this trend. In the present study, we successfully identified the pathogen to the species level using both Biotyper and Vitek MS systems. In terms of the reported cases, four (4/8) was identified on the Bruker Biotyper system and one infection (1/8) was diagnosed using Vitek MS system. Only two cases (2/8) were investigated using 16S rRNA gene sequence. *H. huttiense* infections were usually associated with risk factors, such as pneumonia, hematological system disease and cardiovascular disease. Our study showed that *H. huttiense* infection was associated with cardiovascular disease. In the reported studies, most of *H. huttiense* were isolated from blood, as in the present study.

The cut-off points for the interpretation of *H. huttiense* MICs were essentially in line with the CLSI recommendations for Gram-negative non-fermenters or non-enterobacteriaceae. Güngör et al. (2020) observed that *H. huttiense* was sensitive to teicoplanin, ceftazidime and meropenem. Hernández et al. (2019) reported that *H. huttiense* was sensitive to levofloxacin, ceftazidime, trimethoprim-sulfamethoxazole, minocycline and meropenem and resistant to amikacin and colistin (Hernández et al., 2019; Güngör et al., 2020). Currently, neither CLSI nor EUCAS provided a definite breakpoint for *H. huttiense* and its antibiotic sensitivity test was difficult to perform. In our research, piperacillin-tazobactam, imipenem, meropenem and levofloxacin were found to be effective against *H. huttiense* which was consistent with the results of Güngör ^‘^s and Hernández ^‘^s. Antibiotic susceptibility could serve as a means for differentiating *H. huttiense* from *Burkholderia cepacia complex* as the latter was usually multidrug-resistant, whereas the former was not (Berardino et al., 2019).Therefore, there was no definitive consensus reached on the precise antibiotic therapy for this infection and empirical therapy played an important role in the clinical treatment of *H. huttiense* infections. The publications showed that meropenem and piperacillin/tazobactom were the most commonly used clinical drugs and had good clinical effects. Our patient demonstrated a good clinical response due to the treatment with meropenram and tigecycline, followed by moxifloxacin and piperacillin/tazobactam. Of the 50 virulence associated genes detected in all genomes, several categories of flagellum-associated proteins were detected. Flagellar movement could enhance the invasion of bacteria to the host, because the movement was often chemically oriented and thus could avoid harmful environments or move toward the direction of high concentration environments. It was possible that the virulence of *H. huttiense* was flagella-related. Of the 69 antibiotic resistance genes identified, multidrug-resistance associated genes predominated, suggesting that antibiotic resistance of *H. huttiense* might be due to the possession of these multidrug-resistance associated genes.

Many environmental microorganisms have evolved into human pathogens, and *H. huttiense* is one of these. In the past, the isolation *H. huttiense* had proved esspecially challenging as it was easily be misidentified due to its phylogenetic and phenotypic resemblance to other strains. VITEK 2 and other biochemical identification systems had been unable to identify *H. huttiense.*. *H. huttiense* was frequently confused with organisms such as *B. cepacia complex*, *Cupriavidus pauculus, Ralstonia* spp.*, or Ochrobactrum anthropic.* These limitations had retarded the investigation and knowledge of *H. huttiense*. However, the recent wide establishment of MALDI-TOF MS in clinical microbiology had resulted in the identification of bacteria and fungi with an accuracy of 90% or higher. MALDI-TOF MS was a spectroscopic method which required a reliable and complete database. The prompt (less than 1 h) identification and high discriminatory power of MALDI-TOF MS made it a useful tool for the characterization of rare bacteria that were previously difficult to identify using routine methods. In addition, the detection probability of rare bacteria was improved by the application of bioMérieux MS scientific research database. In our research, MALDI-TOF MS was used to identify an isolated strain of *H. huttiense* with a confidence of 99.2% in the scientific research database. This is the first report of the identification of *H. huttiense* in China by MALDI-TOF MS technology. The 16S rRNA gene sequencing and NGS were used to verify the MALDI-TOF results and a phylogenetic tree was constructed to confirm the findings. The results of MALDI-TOF MS, 16S rRNA gene sequencing, and NGS were consistent, and achieved high accuracy.

## Conclusions

In conclusion, *H. huttiense* was isolated from one clinical sample and identified by MALDI-TOF MS, 16S rRNA gene sequencing and NGS. MALDI-TOF MS and 16S rRNA gene sequencing represented prompt and accurate detection methods and were completed within 24 h. The isolates had good antibiotic activities to imipenem, meropenem, piperacillin-tazobactam and levofloxacin. This demonstration of the prompt identification of a rare pathogen and its antibiotic activities might increase awareness of these uncommon infections.

## Data Availability Statement

The datasets for this article are not publicly available due to concerns regarding participant/patient anonymity. Requests to access the datasets should be directed to the corresponding authors.

## Ethics Statement

The studies involving human participants were reviewed and approved by the Local Research Ethics committee of the First Affiliated Hospital of Anhui Medical University (Quick-PJ2022-02-14). The patients/participants provided their written informed consent to participate in this study.

## Author Contributions

XL, XB, GQ, LW, CS, SC, YX, MZ, and ZW conceived and designed the study. YX, MZ, and ZW were responsible for data interpretation. XL and XB wrote the paper. YX, MZ, and ZW revised subsequent versions. GQ, LW, CS, and SC carried out the experimental works in the clinical microbiology laboratory. All authors have read and approved the final manuscript.

## Funding

This work was supported by Youth Project of National Natural Science Foundation of China (82100613), the Opening Project of Anhui Province Key Laboratory of Reproductive Health and Genetics, Doctoral Research Foundation of the First Affiliated Hospital of Anhui Medical University (Bsky2019038), Scientific Research Fund of Anhui Medical University (2021xkj137) and Anhui Provincial Key Research and Development Plan Project (201904a07020049). The grant number of the Opening Project of Anhui Province Key Laboratory of Reproductive Health and Genetics should be added and the grant number is 9021701201.

## Conflict of Interest

The authors declare that the research was conducted in the absence of any commercial or financial relationships that could be construed as a potential conflict of interest.

## Publisher’s Note

All claims expressed in this article are solely those of the authors and do not necessarily represent those of their affiliated organizations, or those of the publisher, the editors and the reviewers. Any product that may be evaluated in this article, or claim that may be made by its manufacturer, is not guaranteed or endorsed by the publisher.
